# A Smartphone App (AfyaData) for Innovative One Health Disease Surveillance from Community to National Levels in Africa: Intervention in Disease Surveillance

**DOI:** 10.2196/publichealth.7373

**Published:** 2017-12-18

**Authors:** Esron Daniel Karimuribo, Eric Mutagahywa, Calvin Sindato, Leonard Mboera, Mpoki Mwabukusi, M Kariuki Njenga, Scott Teesdale, Jennifer Olsen, Mark Rweyemamu

**Affiliations:** ^1^ Department of Veterinary Medicine and Public Health College of Veterinary Medicine and Biomedical Sciences Sokoine University of Agriculture Morogoro United Republic Of Tanzania; ^2^ Southern African Centre for Infectious Disease Surveillance Sokoine University of Agriculture Morogoro United Republic Of Tanzania; ^3^ National Institute for Medical Research Dar es Salaam United Republic Of Tanzania; ^4^ Kenya Medical Research Institute Nairobi Kenya; ^5^ Innovative Support to Emergencies Diseases and Disasters San Francisco, CA United States; ^6^ Skoll Global Threats Fund San Francisco, CA United States

**Keywords:** public health, surveillance systems, epidemics, outbreak, technology

## Abstract

**Background:**

We describe the development and initial achievements of a participatory disease surveillance system that relies on mobile technology to promote Community Level One Health Security (CLOHS) in Africa.

**Objective:**

The objective of this system, Enhancing Community-Based Disease Outbreak Detection and Response in East and Southern Africa (DODRES), is to empower community-based human and animal health reporters with training and information and communication technology (ICT)–based solutions to contribute to disease detection and response, thereby complementing strategies to improve the efficiency of infectious disease surveillance at national, regional, and global levels. In this study, we refer to techno-health as the application of ICT-based solutions to enhance early detection, timely reporting, and prompt response to health events in human and animal populations.

**Methods:**

An EpiHack, involving human and animal health experts as well as ICT programmers, was held in Tanzania in 2014 to identify major challenges facing early detection, timely reporting, and prompt response to disease events. This was followed by a project inception workshop in 2015, which brought together key stakeholders, including policy makers and community representatives, to refine the objectives and implementation plan of the DODRES project. The digital ICT tools were developed and packaged together as the *AfyaData* app to support One Health disease surveillance. Community health reporters (CHRs) and officials from animal and human health sectors in Morogoro and Ngorongoro districts in Tanzania were trained to use the *AfyaData* app. The *AfyaData* supports near- to real-time data collection and submission at both community and health facility levels as well as the provision of feedback to reporters. The functionality of the One Health Knowledge Repository (OHKR) app has been integrated into the *AfyaData* app to provide health information on case definitions of diseases of humans and animals and to synthesize advice that can be transmitted to CHRs with next step response activities or interventions. Additionally, a WhatsApp social group was made to serve as a platform to sustain interactions between community members, local government officials, and DODRES team members.

**Results:**

Within the first 5 months (August-December 2016) of *AfyaData* tool deployment, a total of 1915 clinical cases in livestock (1816) and humans (99) were reported in Morogoro (83) and Ngorongoro (1832) districts.

**Conclusions:**

These initial results suggest that the DODRES community-level model creates an opportunity for One Health engagement of people in their own communities in the detection of infectious human and animal disease threats. Participatory approaches supported by digital and mobile technologies should be promoted for early disease detection, timely reporting, and prompt response at the community, national, regional, and global levels.

## Introduction

### Background

A growing body of evidence shows that infectious diseases have significant negative socioeconomic consequences on vulnerable populations across the world [1-5]. The impact is enormous in most low- and middle-income countries (LMICs) in sub-Saharan Africa, where capacity for risk management of emerging and reemerging diseases is inadequate, thereby posing challenges to both human and livestock health systems. Infectious diseases account for approximately 40% to 50% of global morbidity and mortality in humans [1], with LMICs recording higher proportions of infectious disease contributions to morbidity and mortality compared with high-income countries [2,3]. It has been recognized that approximately 70% of emerging diseases of humans have an animal origin [4]. Furthermore, infectious diseases in animals constitute a major constraint to livestock-dependent livelihoods and are the single most important barrier to the export of African livestock commodities to lucrative markets [5,6]. These observations, together with increasing international movement of people and commodities, alarming increase in antimicrobial resistance, as well as climate variability or change, emphasize the need for a One Health approach to strengthen risk management of infectious diseases in LMICs in Africa.

Response to infectious disease epidemics largely depends on appropriate and effective surveillance programs that inform both human and animal health decision making and practice. The current disease surveillance systems and strategies in Tanzania are based on the International Health Regulations (IHR 2005) and the World Organization for Animal Health (OIE), which mandate the flow of information from the community to the global level [7]. However, existing systems have been performing suboptimally [8,9]. This raises the question of whether participatory engagement of local communities improves the performance of disease surveillance systems.

The fact that disease outbreaks typically erupt in communities, that is, at the local level, suggests that communities are a key driver influencing the persistence and transmission dynamic of infectious diseases. This is especially true among pastoralists and poor rural communities [10-13]. Moreover, the most vulnerable communities are typically located in remote areas that are hard to reach and that do not have reliable communication which calls for utilization of innovative approaches for early detection and reporting of disease events in near-real time. Some initiatives have been made to facilitate collection of health data in the locations without the Internet. For instance, WeFarm, which is a free peer-to-peer service that enables farmers to share information via short message service without the Internet, has been reported to be useful to support farmers to ask questions on farming and receive crowdsourced prompt responses from other farmers around the world [12]. Another example is Cojengo from a Scotland-based technology company that developed a disease surveillance tool, which is branded as *VetAfrica app*, to help farmers expedite diagnosis of livestock diseases and provide suitable drugs for farm animals [13].

### Opportunities for Strengthening Disease Surveillance

Community-based disease surveillance strategies have the potential to benefit from improved data quality and access, given the current increased trend in the penetration of smartphones and ownership, as well as universal Internet access by rural communities. The use of paper-based system to record and submit health events data in resource-poor countries contributes enormously to delayed response. It is also common practice in African cultures that the health care pathway does not start off at official health facilities but rather at home or traditional healers. Thus, most health events within communities are not captured in the official health surveillance system. The quest for an early warning system calls for community members to be directly involved in the surveillance and detection of health events (ie, participatory epidemiology). Innovative solutions are therefore needed to bridge the gap of capturing health events at community level that should inform the relevant authorities to provide appropriate responses in a timely manner. A disease surveillance approach that not only is grounded in One Health principles but is also participatory, supporting sharing of health information among stakeholders is likely to enhance early detection of human and animal diseases at the community level by empowering communities to take ownership and control over local decisions and to have a stake in maintaining the surveillance structures and practices [12].

The widening use of mobile phones in sub-Saharan Africa, where the penetration rate has reached 67% [14], offers the opportunity to develop innovative participatory surveillance strategies that rely on the design and deployment of digital and mobile technology solutions. In this paper, we describe the Southern African Centre for Infectious Disease Surveillance’s (SACIDS) experience in implementing a participatory surveillance system that relies on digital and mobile technology solutions through the Enhancing Community-Based Disease Outbreak Detection and Response in East and Southern Africa (DODRES) project. In this study, we refer to techno-health as the application of information and communication technology (ICT)–based solutions to enhance early detection, timely reporting, and prompt response to health events in human and animal populations. The DODRES project is supported by Skoll Global Threats Fund. Its overall goal is to promote Community Level One Health Security (CLOHS), thus complementing international disease surveillance strategies with participatory engagement of local communities and enhancing early disease detection and response at community, national, regional, and global levels.

## Methods

### Development and Implementation of Innovative Ideas to Strengthen Disease Surveillance

The DODRES project is a part of SACIDS’ continuing efforts to champion a CLOHS initiative to support participatory approaches in disease surveillance that complement the Global Health Security Agenda. The Global Health Security Agenda is a partnership of nearly 50 nations, international organizations, and nongovernmental stakeholders that was launched in February 2014 to help build countries’ capacities to create a world that is safe and secure from infectious disease threats and to elevate global health security as a national and global priority [15,16].

SACIDS held two events to promote CLOHS and lay the groundwork for the DODRES project. The first was EpiHack Tanzania, which was held in Arusha, Tanzania, in December 2014. EpiHack is a collaborative gathering of software developers and health professionals to create digital technological solutions that address specific public and animal health issues. The aim of EpiHack Tanzania was to bring together experts from the animal and human health sectors, as well as ICT developers to collaborate in providing solutions to challenges facing infectious disease surveillance and response in the Southern and Eastern African regions. The second event was a project inception workshop held in August 2015, also in Arusha, Tanzania. The workshop was organized to bring together key stakeholders considered to be important for successful implementation of the project. In the two events, it was agreed to promote CLOHS through (1) enhancing working across animal and human sectors to fight epidemics in human and animal populations; (2) developing ICT tools to support data capture, reporting, and feedback at health facilities and within communities that feed into the official Integrated Disease Surveillance and Response (IDSR) and veterinary national surveillance systems; and (3) strengthening local cross-border collaboration to fight epidemics.

In November 2015, the Techno-Health Innovative Laboratory was established at Morogoro Regional Hospital in Morogoro, Tanzania, to host the DODRES design and implementation team of epidemiologists and ICT programmers. While the SACIDS-National Institute for Medical Research (NIMR) Design and Implementation team led ICT tool development, the US-based Innovative Support to Emergencies, Diseases, and Disasters (InSTEDD) provided mentorship and quality assurance of the tools developed. Subsequently, project sites for piloting DODRES were selected. A theory of change (ToC) framework was used to guide the DODRES project implementation process. A ToC refers to a tool that is used to hypothesize on how and why an initiative works [17]. It is a systematic and cumulative study of the links between activities and inputs, outcomes, and contexts of the initiative. The ICT team developed four prototype ICT tools and then implemented three finalized ICT tools (packaged together as the *AfyaData* app) for near real-time participatory data collection, reporting, and feedback. SACIDS trained One Health community health reporters (CHRs) and facility and district officials to use the new tools. Each of these activities is described in detail below.

### Description of the Project Sites

**Figure 1:**
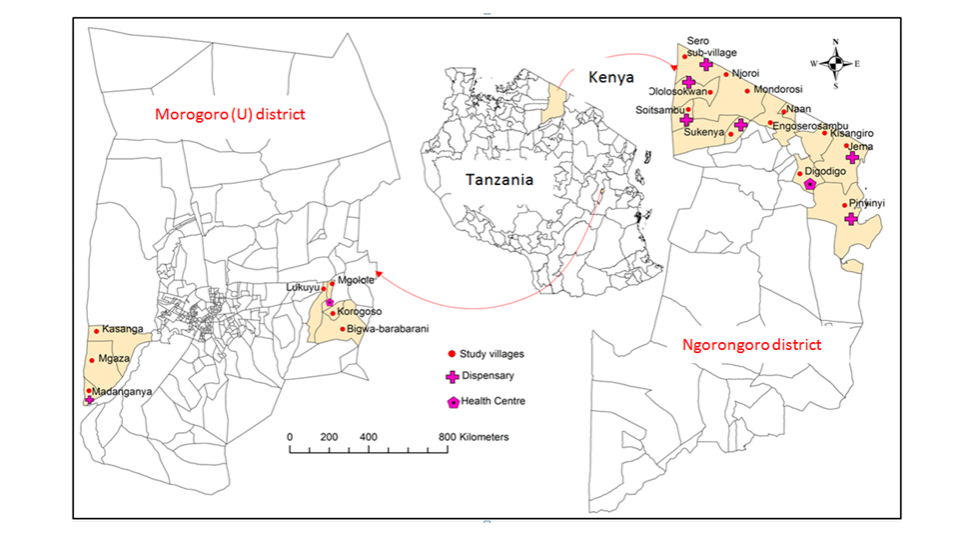
Map showing DODRES project sites in the Morogoro Urban and Ngorongoro districts of Tanzania.

Two project sites were strategically selected for the piloting of DODRES project (Figure 1). One was the Ngorongoro district of Tanzania and Narok County in Kenya. The two districts share the same cross-border ecosystem. This inland ecosystem is not only contiguous with the major wildlife ecosystems of the Ngorongoro Conservation Area Authority, Serengeti National Park, and Maasai Mara Wildlife Reserve but is also characterized by maximum informal interactions of the local pastoral (Maasai) community in both Tanzania and Kenya. Thus, human-domestic-wildlife interactions are frequent, and the area is at high risk for both human and animal disease epidemics, including Rift Valley fever (RVF, 2006/2007), contagious bovine pleuropneumonia (CBPP, 2010-2012), contagious caprine pleuropneumonia (CCPP), peste des petits ruminants (PPR, 2008 to date) [18-20], and anthrax (Ngorongoro District Council, unpublished). The area is inhabited by an estimated 1,025,198 people and 3,458,027 livestock (ie, cattle, goats, and sheep). The total land area of this ecosystem is approximately 31,957 square kilometers. SACIDS has plans to also deploy *AfyaData* tools in Narok County in Kenya to strengthen local cross-border collaboration.

The second site was the Morogoro Urban district in central-eastern Tanzania. This district is inhabited by 602,114 people occupying approximately 260 square kilometers. It was strategically selected to participate in DODRES project implementation because the core project design and implementation team is hosted within the Morogoro Urban district. Thus, ICT tools can be more readily tested in proximity to where the ICT team is housed before being deployed for field data collection and reporting in other project sites.

### Theory of Change for Guiding DODRES Implementation

**Figure 2:**
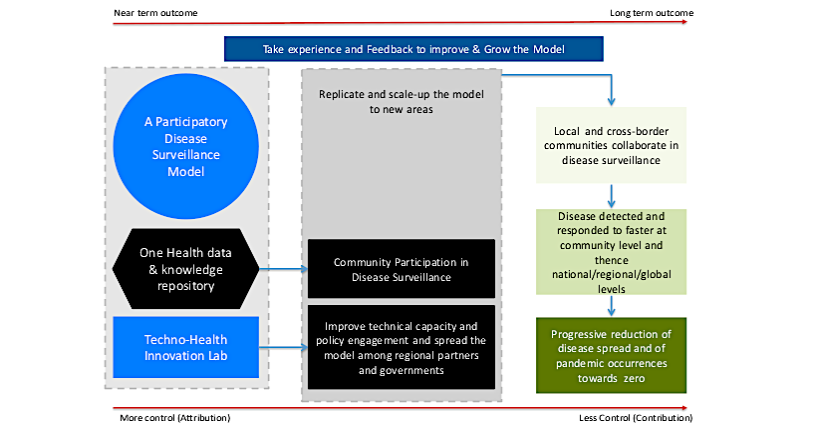
Community Level One Health Security theory of change.

A ToC (Figure 2) was developed to guide DODRES project implementation. The ToC is a tool normally developed to guide planning, participation, and monitoring and evaluation of a given project that aims at bringing social change.

Key components of the ToC are as follows: (1) a participatory disease surveillance model; (2) improvement and growth of the model; and (3) long-term contribution to progressive reduction of disease spread and of pandemic occurrences at the community, national, and global levels. It was perceived that, as a first step to achieving its ultimate goal, establishing the Techno-Health Innovation Laboratory to support the design of participatory disease surveillance tools and the One Health Knowledge Repository (OHKR) were processes that were within the ability of DODRES project to plan and implement. It was further considered that implementation of both processes would improve community participation in disease surveillance and produce evidence to influence policy change with respect to disease surveillance in both the human and animal health sectors. The ultimate goal of the DODRES project is to contribute to the reduction of infectious disease spread and pandemic occurrences toward zero prevalence—an achievement that requires joint efforts with other actors and less control by the DODRES project.

The ToC was therefore developed using participatory approach involving human and animal health experts in collaboration with the ICT experts. This ToC is centered on the theory of promoting use of participatory disease surveillance, ICT tools, and application of One Health collaborative approaches as key inputs/activities to support early disease outbreak detection and response (outputs) (Figure 2). The outcomes of such an initiative, which are contributed to by the DODRES project in partnership with other stakeholders, include faster detection of infectious diseases at different levels (community, national, regional, and global) that consequently add to progressive reduction of disease spread and pandemic occurrences globally. The ToC was used to guide development of potential One Health participatory disease surveillance technological solutions, taking into consideration the main challenges to effective infectious disease surveillance that were identified during EpiHack Tanzania and at the 2015 project inception workshop. These challenges and technical solutions are outlined in Figure 2.

### Challenges to Disease Surveillance

Through a participatory problem identification process, the following list of key challenges was jointly developed by ICT programmers and human and animal health experts:

- Failure to capture major disease events occurring at the community level because of application of traditional nonparticipatory approaches in public and animal health disease surveillance
- Delayed submission and incompleteness of official disease surveillance data submitted by health facilities to the subnational and national levels
- Lack of feedback (two-way communication) to the disease surveillance data collectors
- Inability to trace individual humans and animals, as well as their locations, during disease outbreaks

### Recommended Technical Solutions

The ICT programmers, in collaboration with human and animal health experts, designed the following four prototype technological solutions to address these challenges:

1. Community-based participatory disease surveillance for timely detection and reporting of disease events at the community level
2. Official surveillance strategy for timely collection and submission of disease data at the health facility level
3. Two-way communication feedback loops to provide prompt feedback and hence value to individuals who report disease events at community and health facility levels
4. Contact tracing, including identification of affected households or livestock herds and their locations, to support official tracking of potential disease outbreaks and to aid outbreak investigation

### Development of ICT Tools to Support Participatory Disease Surveillance

**Figure 3:**
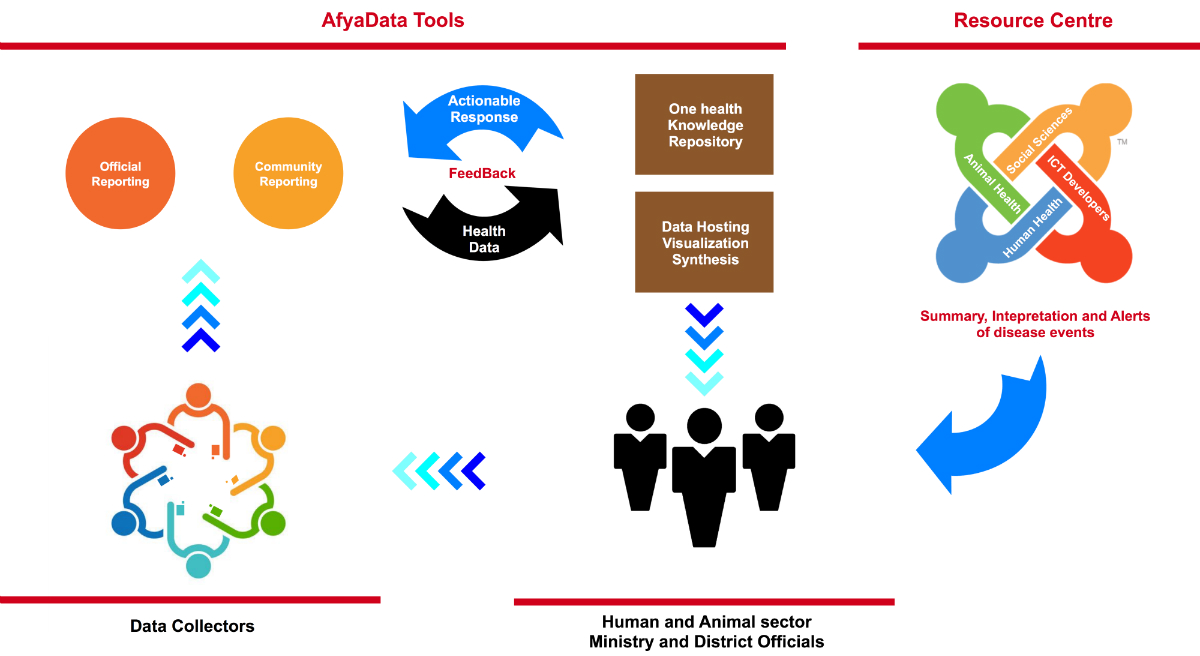
Integrated development of information and communication technology (ICT) tools (the AfyaData app) to support data collection and feedback on disease events occurring in human and animal populations.

From the abovementioned prototypes, three (1, 2, and 3) were integrated into an *AfyaData* app (Figure 3) and developed into a stable beta version to support technical solutions for near real-time data collection at community and health facility levels and for the provision of feedback to reporters. *AfyaData* is a set of two apps; a native mobile Android-based client and a Web-based app acting as a server. The mobile client is inspired by Open Data Kit (ODK), used for collecting and submitting syndromic data and receiving and/or tracking feedback from health officials. The server component consists of a set of Web service that handles the entire lifecycle for initializing, collecting, registering, and managing forms ready for the *AfyaData* mobile client to utilize.

The system is designed to collect data using a mobile phone with or without Internet connectivity, and data can be submitted at a location with the Internet. Source code is hosted on GitHub [21], manufactured by GitHub, Inc.One of the most common challenges in the traditional infectious disease surveillance systems in human and animal health is a lack of timely feedback. In particular, feedback to persons collecting and submitting surveillance data is critical to encourage them to continue reporting quality data, as well as initiate quick actions to prevent or mitigate the extent of possible epidemics. Having recognized the importance of prompt detection, reporting, and feedback, an OHKR module was developed as an integral component of the *AfyaData* app hosted on the server at the Sokoine University of Agriculture. To start with, SACIDS has engaged specialists in the fields of human and animal health to develop OHKR contents for priority endemic and epidemic-prone diseases in East Africa. The source code is open source, and data are accessible to ascribed stakeholders.

The OHKR is a decision-making system with expert-authored content that helps to support the prediction of likely disease conditions based on signs and symptoms reported by CHRs, thereby guiding confirmation of diseases. The contents of OHKR include specific disease standard case definition, percentage weight of clinical signs for each disease, and answers to frequently asked questions. The specialists from human and animal health sectors assigned percentage weight to each clinical manifestation associated with specific disease based on the extent to which such a clinical manifestation explains the disease. For instance, in cattle, the presence of blisters on snout, tongue, and space between hoofs (interdigital space) would explain about 80% chances of the disease being foot-and-mouth disease (FMD), whereas salivation, fever, and sudden onset of lameness would explain about 40% chances of the disease being FMD. The OHKR has been programmed to predict the most likely disease condition based on these percentage weights to inform subsequent strategic investigation/confirmation. We report herein the preliminary performance of the system, and plans are under way to validate it. The OHKR comprises three subsystems: (1) content subsystem—expert-authored content; (2) score map subsystem—symptom scores, weighted with respect to a particular disease, species, and location; and (3) turnkey mapper subsystem—a three-dimensional iterative matching algorithm. The OHKR system initiates its process as it receives clinical manifestations from a newly reported case/incident in AfyaData mobile app. These data are cleaned, combined, and compared with a preset clinical manifestation scores map. Weighted scores are cumulatively added for each combination of clinical manifestation received for the particular disease it matches with. Relevant content of the most likely diseases based on the cumulative score is then retrieved and fed into the feedback system. A list of recommended actions has been created per targeted user (community health workers/reporters, livestock extension officers, in-charge of health facilities, and district medical/veterinary officers). The OHKR automatically sends messages to relevant user on artificial intelligence and alerts of possible disease conditions occurring in human and animal populations.

### Training and Support of Community Health Reporters and Officials

With funding from the Rockefeller Foundation (2010-2012) and the Canadian International Development Research Centre (IDRC) (2013-2017), between 2010 and 2016, SACIDS trained and empowered 82 frontline One Health CHRs, 41 facility-based health officials, 33 livestock field officers, and 14 data managers and analysts based at district medical and veterinary offices in Tanzania, Zambia, Burundi, and Kenya. During this period, the open source EpiCollect and ODK apps were used to sustain mobile-supported disease surveillance at health facility and community levels. The DODRES project built on this work, training an additional 29 CHRs and 17 officials in Morogoro Urban and Ngorongoro districts in early disease detection, reporting, and response. With DODRES, SACIDS changed its training policy by supporting more reporters (CHRs) at the community level, rather than at the health facility level (ie, official), to increase the likelihood of capturing events at the community level. Additionally, in remote areas where there is no alternative power source, the DODRES project provided the CHRs with solar chargers to ensure that their mobile phones remain powered.

The health data collected by CHRs and associated geographical coordinates are submitted to a centralized server system that supports near real-time access to all ascribed stakeholders. The spatial distribution of health events was created on ArcGIS 10.4.1. The latitude and longitude coordinates were projected onto the map using the World Geodetic System 1984 datum. In addition to the CHRs receiving feedback after submitting health events data, the *AfyaData* system provides a two-way interaction between the CHR and the health specialists to inform appropriate actions. The established WhatsApp social networking serves as a *crowdsourcing* platform that provides opportunity to share best practices, challenges, and solutions among CHR themselves and between CHRs and health specialists from animal and human health sectors.

Since March 2016, the DODRES project has also engaged ICT developers, health experts, and policy makers through monthly publication of the *TechnoHealth Surveillance* newsletter. The newsletter is used as a channel to disseminate disease surveillance information among project partners and the general public. The mailing list that receives the newsletter currently stands at 350. Additionally, all trained CHRs are networked with district health experts and DODRES project team members via the WhatsApp social platform. The WhatsApp networking not only contributes to sustained interactions between community members, government officials, and DODRES team members, but it also provides unstructured real-time information, thereby complementing the feedback mechanism tool of the *AfyaData* app. Using the same networking, health experts can provide instant technical support and advice on community-level outbreak management.

### Ethics Statement

This study was approved by the Tanzania Medical Research Coordinating Committee of the National Institute for Medical Research (NIMR/HQ/R.8a/Vol.IX/2037).

## Results

### Disease Syndromes Reported

Of the 1915 clinical cases in livestock (1816) and humans (99) reported by CHRs using *AfyaData* app from August 2016 to December 2016, 95.98% (1838/1915) and 4.02% (77/1915) cases were from Ngorongoro and Morogoro Urban districts, respectively. Overall, a total of 1816 livestock cases were reported from a total population of 12,114 animals, of which 867 died, translating to a population morbidity rate and case fatality rate (CFR) of 14.99% (1816/12,114) and 47.74% (867/1816), respectively. The Ngorongoro animal population included 7613 goats from 45 flocks, 1948 cattle from 26 herds, 2250 sheep from 8 flocks, 144 chickens from 2 flocks, and 14 dogs from 2 kennels. The animal population in Morogoro included 5 goats from 1 flock, 6 cattle from 1 herd, 115 chickens from 4 flocks, and 13 dogs from 1 kennel. Out of 1816 livestock cases, 1750 were reported in Ngorongoro, whereas 66 out of 1816 livestock cases were reported in Morogoro. On the other hand, 82 out of 99 human cases were reported in Ngorongoro, whereas 17 out of 99 human cases were reported in Morogoro. Out of 1816 animal cases, 1762 were reported in domestic ruminants (goats, cattle, and sheep), whereas 54 out of 1816 cases were reported in chicken, pigs, and dogs. Out of 1826 animal cases, 1053 were reported in goats. Other livestock cases were reported in sheep (435), cattle (253), chicken (73), and dogs (2). Of 1816 livestock cases, 1736 were reported in domestic ruminants, of which 1042, 434, and 260 were goats, sheep, and cattle, respectively. Out of 1736 domestic ruminant cases, 1180 were aged ≥1 year. Generally, there was a tendency for the number of domestic ruminant cases and associated CFR to increase over subsequent months from 79 cases (CFR=6% [5/79]) reported in August 2016 to 793 cases (CFR=54% [431/793]) reported in December 2016.

**Figure 4:**
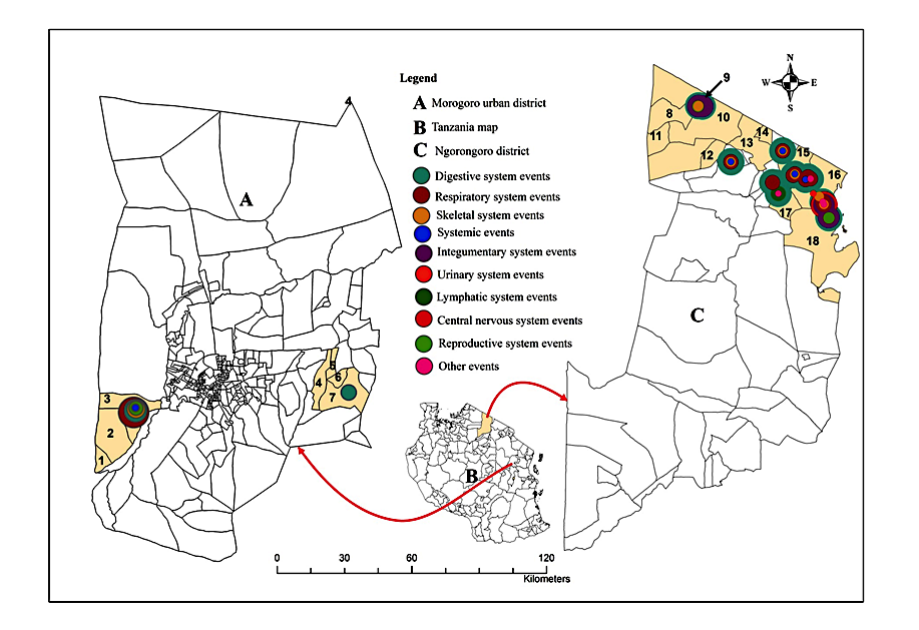
Spatial distribution of major clinical syndromes reported in livestock population in Morogoro Urban and Ngorongoro districts in Tanzania.

The spatial distribution of syndromes reported in animals in the two districts is shown in Figure 4. Overall, the most frequently reported clinical manifestations were related to the respiratory system (ie, coughing, rapid breathing, sneezing, difficulty breathing, and nasal discharge; 159 reports), digestive system (ie, loss of appetite, diarrhea, frothy discharge from mouth, bloody diarrhea, and lesions in the mouth; 93 reports), reproductive system (ie, reduced milk production and abortion; 55 reports), and systemic disease (ie, fever and bleeding from natural body openings; 43 reports). Other reported manifestations were related to the nervous system (ie, twisted neck, circling, and abnormal behavior; 18 reports) and integumentary system (ie, swollen joints; 5 reports). Clinical manifestations such as reluctance to walk (36 reports) and discharge from eyes (19 reports) were also reported.

Using chi-square test, the overall population morbidity rate was significantly higher for domestic ruminants aged <1 year (30.21%, 564/1867) than those aged ≥1 year (11.77%, 1172/9955; *P*<.05). The CFR in the animals aged <1 year and ≥1 years were 46.6% (263/564) and 47.35% (555/1172), respectively. (*P*=.08). The population morbidity rate varied significantly by month for domestic ruminants aged ≥1 year, with higher values recorded in December (21%) and November (16%; *P*<.05). The CFR also varied significantly with calendar month in domestic ruminants aged ≥1 year, with similarly higher values been recorded in November (61.0%, 213/349) and December (50.7%, 262/517; *P*<.05). In addition, the population morbidity rate for domestic ruminants aged ≥1 year varied significantly among the study villages, with higher values been recorded in Bigwa-Barabarani (100%, 3/3), Naan (35.00%, 353/1463), Sukenya (29%, 17/58), and Kindibwa (25%, 1/4) villages (*P*<.05).

**Figure 5:**
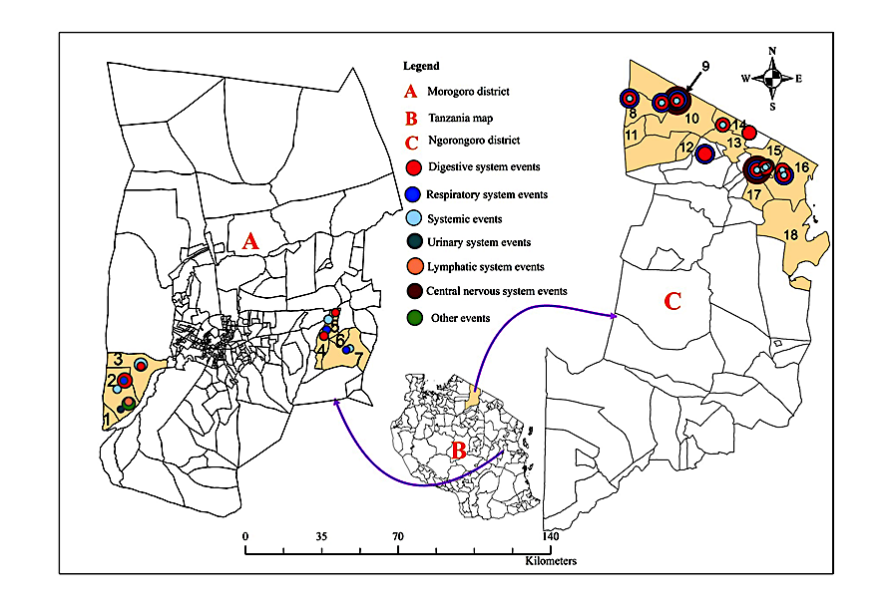
Spatial distribution of major clinical syndromes reported in human population in Morogoro Urban and Ngorongoro districts in Tanzania.

The spatial distribution of syndromes reported in humans in the two districts is shown in Figure 5. A total of 99 human cases were reported in 16 out of 18 study villages, with significantly larger number of cases (82) being from 10 of the 11 study villages in Ngorongoro compared with 17 cases being reported in 6 out of 7 study villages in Morogoro Urban district (*P*<.001). The majority of human cases reported in Ngorongoro (n=82) were from Kisangiro (36 cases), followed by Ololosokwan (18 cases), Jema (11 cases), Njoroi (5 cases), and Pinyinyi (5 cases) villages. Other human cases in the district were reported in Naan (4 cases), Enguserosambu, Mondorosi, Soitsambu, and Sukenya (1 case each) villages. In Morogoro Urban district, human cases (n=17) were reported from Kasanga (7 cases), Bigwa-Barabarani (3 cases), Mikoroshini (3 cases), Chamwino (2), and Lukuyu and Mgaza (1 case each). Overall, out of 99 human cases, 56 were reported among males, and 68 cases were reported among individuals aged ≥5 years. Out of 31 cases among individuals aged <5 years, 10 were reported in October, whereas 19 out of 68 cases among individuals aged ≥5 years were reported in August.

Overall, the most frequently clinical manifestations reported among humans in both districts were those related to the digestive system (104), including loss of appetite (27), diarrhea (24), vomiting (18), stomach ache (17), constipation (6), bloody vomiting (5), bloody diarrhea (4), and lesions in the mouth (3); the values in parenthesis represent the number of clinical manifestations. The next most frequently reported clinical manifestations were related to the respiratory system (56), including coughing (38), difficulty breathing (8), rapid breathing (6), and bloody coughing (4). The most frequently reported clinical manifestations among humans in Ngorongoro included coughing (34), headache (30), loss of appetite (22), diarrhea (18), body weakness (15), fever (14), vomiting (13), and stomach ache (13).

On the basis of the clinical manifestations reported, the most probable infectious conditions identified in goats by OHKR, with likelihood percentages in parentheses, were PPR (90%) and CCPP (80%). The most probable infectious diseases in cattle were CBPP (50%), brucellosis (50%), and anthrax (30%). The most probable disease in dogs was rabies (90%), whereas those in humans were malaria (65%), cholera (60%), and anthrax (30%).

### Lessons Learned During Implementation

Since 2010, SACIDS-managed initiatives to utilize participatory approaches and mobile technologies have been contributing to improved disease surveillance in both human and animal populations in East and Southern African regions.

The DODRES ICT programming team has worked in close collaboration with the epidemiology team to design and deploy ICT tools that are relevant to disease surveillance in both the public and animal health sectors. An important lesson learned during the implementation of these various SACIDS projects, including DODRES, is the importance of making use of local expertise to design and maintain ICT-supported systems. The local team provided immediate technical support and was found to be reliable. In all of these projects, open-source apps have been used. Maintaining an open-source policy allows for contributions to app development by the open-source communities and provides a means of technical support for designing, testing, and refining newly developed ICT tools.

A baseline field assessment conducted by SACIDS in 11 villages in Ngorongoro and 7 villages in Morogoro Urban districts in April 2016 to establish benchmark values for performance indicators measured throughout DODRES project implementation indicated that CHRs and field-based health officials in the project sites considered mobile technologies to be more useful than paper-based systems for disease surveillance. Although field workers have run into some challenges, such as failing to synchronize data on their mobile phones with the server, recording geographical coordinates of more than one case at one location, and failing to locate clinical cases in mobile pastoral communities after receiving a call, the ICT developers have helped to correct some of these challenges.

## Discussion

### Principal Findings

Using participatory community-based digital disease surveillance approaches, we recorded a total number of 1915 clinical cases in livestock and humans within the first 5 months of *AfyaData* deployment in Morogoro and Ngorongoro districts.

Initial results of DODRES project show more clinical cases in both humans and livestock captured and reported by CHRs in Ngorongoro than in Morogoro Urban district. This difference may be attributed to lifestyle differences between the two sites. Morogoro is a densely populated urban district where the majority of population can easily access medical and veterinary services. In contrast, Ngorongoro is a rural-based district with sparsely populated villages located far from health facilities, thus making the work of CHRs even more valued than in urban areas. Similarly, the livestock production systems between the two areas differ, with Morogoro Urban district having a backyard zero grazing production system. In contrast, Ngorongoro is dominated by a pastoral production system. Using a participatory approach, CHRs were able to detect and report different clinical cases and identify symptoms related to a broad range of body systems (eg, digestive, respiratory, integumentary, and central nervous).

Utilization of mobile phones and ICT technologies to improve disease surveillance in public and animal health has been reported in other countries, including China [22], Sri Lanka [23], Zambia, Madagascar, Uganda, and Kenya [24]. The use of community health workers in public and animal heath sectors has been piloted in other countries as well [25-27]. A few of these studies or systems have combined the use of mobile technologies with participatory approaches as SACIDS has done. The combination of participatory community-based approaches with mobile technology has the potential to support not only early detections of disease events happening at the community level [28] but also near real-time responses.

By supporting detection of early disease epidemic signals, the DODRES approach has great potential to complement traditional public and animal health surveillance systems as recorded elsewhere [29]. The DODRES project did not support laboratory confirmation of reported disease events; its diagnostic capacity could be enhanced by doing so. Adoption of point-of-care diagnostics in particular, especially in remote areas, would likely hasten confirmation of events where they occur and thereby contribute to timely appropriate management and control of both known and unknown diseases [30]. More studies are needed to evaluate the contribution of community-level approaches to health outcomes, particularly in resource-restricted countries and ecosystems.

### Conclusions

The DODRES model of CLOHS has the potential to contribute significantly to the Global Health Security Agenda by engaging *neglected* members of the community. Participatory approaches supported by mobile technologies should be promoted for enhanced early disease detection and response at the community, national, regional, and global levels.

## Abbreviations

CBPP: contagious bovine pleuropneumonia
CCPP: contagious caprine pleuropneumonia
CHRs: community health reporters
CLOHS: Community Level One Health security
DODRES: Enhancing Community-Based Disease Outbreak Detection and Response in East and Southern Africa
FMD: foot-and-mouth disease
ICT: information and communication technology
IDRC: International Development Research Centre
IDSR: Integrated Disease Surveillance and Response
IHR: International Health Regulations
InSTEDD: Innovative Support to Emergencies, Diseases, and Disasters
LMICs: low- and middle-income countries
NIMR: National Institute for Medical Research
ODK: Open Data Kit
OHKR: One Health Knowledge Repository
OIE: World Organization for Animal Health
PPR: peste des petits ruminants
SACIDS: Southern African Centre for Infectious Disease Surveillance
ToC: theory of change
